# LP99: Discovery and Synthesis of the First Selective BRD7/9 Bromodomain Inhibitor**

**DOI:** 10.1002/anie.201501394

**Published:** 2015-04-13

**Authors:** Peter G K Clark, Lucas C C Vieira, Cynthia Tallant, Oleg Fedorov, Dean C Singleton, Catherine M Rogers, Octovia P Monteiro, James M Bennett, Roberta Baronio, Susanne Müller, Danette L Daniels, Jacqui Méndez, Stefan Knapp, Paul E Brennan, Darren J Dixon

**Affiliations:** Department of Chemistry, Chemistry Research Laboratory, University of Oxford Mansfield Road, Oxford, OX1 3TA (UK); Structural Genomics Consortium & Target Discovery Institute, University of Oxford NDM Research Building, Roosevelt Drive, Oxford, OX3 7DQ and OX3 7FZ (UK); Promega Corporation 2800 Woods Hollow Road, Madison, W153611 (USA)

**Keywords:** bromodomain, cascade reactions, enantioselective catalysis, epigenetics, organocatalysis

## Abstract

The bromodomain-containing proteins BRD9 and BRD7 are part of the human SWI/SNF chromatin-remodeling complexes BAF and PBAF. To date, no selective inhibitor for BRD7/9 has been reported despite its potential value as a biological tool or as a lead for future therapeutics. The quinolone-fused lactam **LP99** is now reported as the first potent and selective inhibitor of the BRD7 and BRD9 bromodomains. Development of **LP99** from a fragment hit was expedited through balancing structure-based inhibitor design and biophysical characterization against tractable chemical synthesis: Complexity-building nitro-Mannich/lactamization cascade processes allowed for early structure–activity relationship studies whereas an enantioselective organocatalytic nitro-Mannich reaction enabled the synthesis of the lead scaffold in enantioenriched form and on scale. This epigenetic probe was shown to inhibit the association of BRD7 and BRD9 to acetylated histones in vitro and in cells. Moreover, **LP99** was used to demonstrate that BRD7/9 plays a role in regulating pro-inflammatory cytokine secretion.

Bromodomains (BRDs) are protein interaction modules that selectively recognize ε-*N*-lysine acetylation motifs, a key event in reading the posttranslational modifications that constitute the epigenetic code. BRD-containing protein 7 (BRD7), which is frequently down-regulated in cancer,^1^ has a proposed tumor suppression function through regulation of p53^2^ and PI3K.^3^ Furthermore, BRD7 has been shown to be required for BRCA1-dependent transcription,^4^ and BRD7 polymorphism has been linked to an increased risk of pancreatic cancer.^5^ In contrast, BRD9 is often over-expressed in cancer owing to a gain of the short arm of chromosome 5 (5p), the most frequent karyotypic change in cervical cancer.^6^ The closely related BRDs BRD7 and BRD9 are part of the SWI/SNF nucleosome-remodeling complex, which plays a crucial role regulating gene expression programs, including the expression of inflammatory genes. Although the functions of the catalytic subunits of this complex, BRM and BRG1, in immune phenomena and inflammatory responses have been described, a role for BRD7 and BRD9 in inflammatory processes has not yet been demonstrated.^7^ Owing to the complexity of BRD7/9-mediated interactions in chromatin, selective, potent inhibitors of these bromodomains would constitute valuable biological tools, enabling functional studies on these essential chromatin interaction domains and potentially allowing for exploitation in small-molecule therapies for various diseases. To date, no potent and selective inhibitors have been reported.^8^

Our aim was to design and develop a probe for the BRD7 and BRD9 bromodomains, achieving potency and selectivity with a suitably decorated fragment^9^ rich in sp^3^-hybridized carbon atoms that, guided by biophysical assays, was designed to maximize specific binding interactions whilst retaining synthetic tractability. Through the use of reaction cascades, which bring together simple starting materials to quickly generate structurally complex products in an efficient one-pot process,^9^, ^10^ we hoped to quickly generate structure–activity relationships (SARs). Subsequently, enantioselective catalysis would allow the scaled synthesis of known enantiomers of late-stage intermediates for advanced SARs, and, ultimately, the scaled-up synthesis of a probe compound.

The development of the first potent and selective BRD7/9 BRD inhibitor began with the simple fragment 1-methylquinolone (**1**), which was shown to be an orthosteric ligand of the BRD of BRD9-related ATAD2 (Figure 1 A).^11^ Compound **1** was also shown to bind BRD9 by isothermal titration calorimetry (ITC; Figure 1 B; see also the Supporting Information, Figure S1). The *N*-methyl amide moiety is an acetyl lysine mimetic, forming similar hydrogen bonds to a conserved asparagine (N1064) and a water molecule as seen in acetyl–lysine recognition. Although BRDs share similar acetyl–lysine recognition motifs, there are significant differences in distal parts of the binding pocket. Owing to a shift in the ZA loop in BRD9, this region is much larger in BRD9, with residues A46, F47, P48, T50, and I53 forming a large hydrophobic cavity (Figure 1 B). The C7 position was viewed as an ideal attachment point for a structurally complex heterocyclic appendage that could exploit this cavity for selective inhibition of BRD9. Furthermore, analysis of the BRD9/**1** model suggested that a C4 methyl group on the quinolone would occupy a shallow hydrophobic pocket, thereby increasing potency.

**Figure 1:**
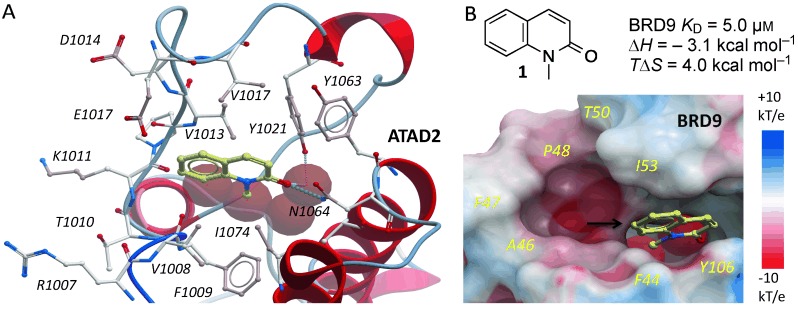
Fragment hit for BRD9. A) 1 (pale sticks) binds ATAD2 BRD via H bonds (dotted lines) to N1064 and conserved water molecules (red spheres; PDB 4QST). B) Electrostatic surface representation of BRD9 overlaid with 1 and conserved water molecules from ATAD2 for reference. The black arrow in (B) indicates the attachment point targeted for the design of selective inhibitors.

To identify the best core scaffold for asymmetric elaboration, a series of quinolones bearing various N-heterocycles were synthesized (Scheme 1).^12^ Compound **4**, readily accessible by known methods,^13^ was used in palladium-catalyzed Buchwald–Hartwig couplings to give various cyclic amides, carbamates, and ureas (**5**–**10**). All compounds were tested for BRD9 BRD binding using a differential scanning fluorimetry (DSF) assay (Table 1). Changes in the melting temperatures (Δ*T*_m_) confirmed that nearly all of the elaborated quinolones were tolerated, and valerolactam **6** was selected as a lead structure for further characterization and optimization. The valerolactam series was selected in preference to the urea series mainly because of the wealth of direct and reliable methods available for their asymmetric synthesis. Co-crystallization with BRD9 showed the conserved H bonding of acetyl–lysine recognition: Compound **6** exhibited H bonds to N100 and to a conserved water molecule. The valerolactam moiety extends into the desired hydrophobic region between F44 and I53 whilst positioning the amide carbonyl near, but not in H bond contact with, Y106. Furthermore, the introduced methyl group at the C4 position demonstrated additional hydrophobic interactions with A54 and Y106. ITC confirmed the activity determined by the DSF assay: **6** bound to BRD9 with a dissociation constant (*K*_D_) of 612 nm (Figure 2 B).

**Figure 2:**
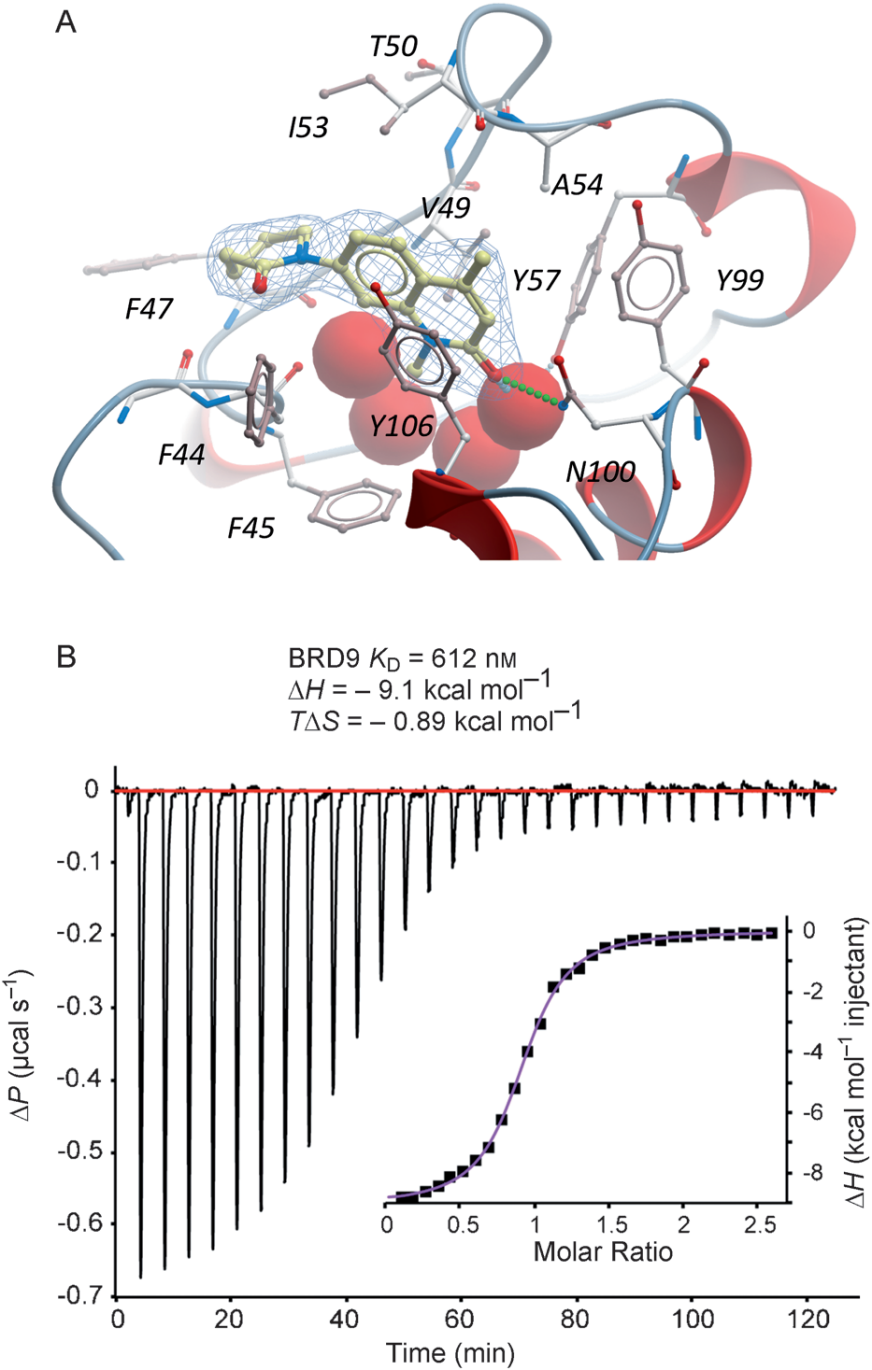
A) Co-crystal structure of compound 6 (yellow sticks) and BRD9 BRD (grey sticks and red ribbon) with the ligand electron density (2FoFc, blue mesh). B) Compound 6 binds to BRD9 with a *K*_D_ value of 612 nm according to ITC analysis.

**Scheme 1:**
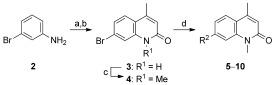
Synthesis of heterocycle-substituted quinolones. a) ethyl acetoacetate, xylenes, reflux, 24 %; b) H_2_SO_4_, 88 %; c) NaH, MeI, DMF, 76 %; d) heterocycle, [Pd_2_(dba)_3_], Xantphos, Cs_2_CO_3_, 1,4-dioxane, 100 °C, 5–96 %. dba=dibenzylideneacetone, Xantphos=4,5-bis(diphenylphosphanyl)-9,9-dimethylxanthene.

**Table 1:** Binding of the substituted quinolones 5–10 to BRD9 bromodomain as determined by DSF Δ*T*_m_ shift.

	Compound	R^2^	BRD9 Δ*T*_m_ [°C]^[a]^	
	**5**	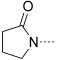	4.0±0.74 (4)	
	**6**	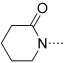	5.9±0.48 (4)	
	**7**	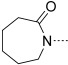	4.4±0.26 (4)	
	**8**	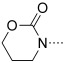	2.5±0.35 (4)	
	**9**	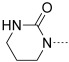	6.1±0.71 (4)	
	**10**	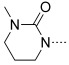	1.6±0.49 (4)	

[a] Mean Δ*T*_m_±SEM (number of measurements). Compounds tested at 10 μm. SEM=standard error of the mean.

SARs for the valerolactam moiety of **6** were rapidly generated through the use of a nitro-Mannich/lactamization cascade process, which also provided a nitro group as a chemical handle for further derivatization. Reactions of 4-nitrobutanoates (**11**, **12**) with ammonium acetate and a range of aldehydes afforded *trans*-5-nitropiperin-2-ones (**13**–**16**) in good yields and modest to excellent diastereoselectivity (Scheme 2).^14^ Reduction of the nitro group with nickel boride, Boc protection of the resulting amines (**17**–**20**), and Buchwald–Hartwig couplings with bromide **4** gave a series of C4/C5/C6-substituted compounds (**21**–**24**), which were tested against BRD9 for potency and BRD4(1) for selectivity by DSF (see Table S1). All of the compounds showed selectivity for BRD9 over BRD4(1); however, lactam **24** (R^1^=H, R^2^=Ph) was considered to be the most promising compound for further development owing to its potency and the opportunity for further optimization. To investigate any stereochemical preferences, **24** was resolved into its component enantiomers by preparative HPLC on a chiral stationary phase. The importance of the absolute stereochemical configuration of the inhibitors was confirmed by the fact that (−)-**24** showed a modest increase in potency compared to **6** (ITC: *K*_D_=493 nm vs. 610 nm) whereas (+)-**24** was an order of magnitude weaker (ITC: *K*_D_=4.3 μm; see the Supporting Information). Co-crystallization of the active enantiomer, which was shown to have a 2*R*,3*S* absolute configuration, with BRD9 (Figure S9) revealed binding consistent with that of **6**, but with additional H bond interactions observed between the NH motif of the Boc-protected amino group to the backbone carbonyl group of G43, and between the lactam carbonyl group to Y106.

**Scheme 2:**
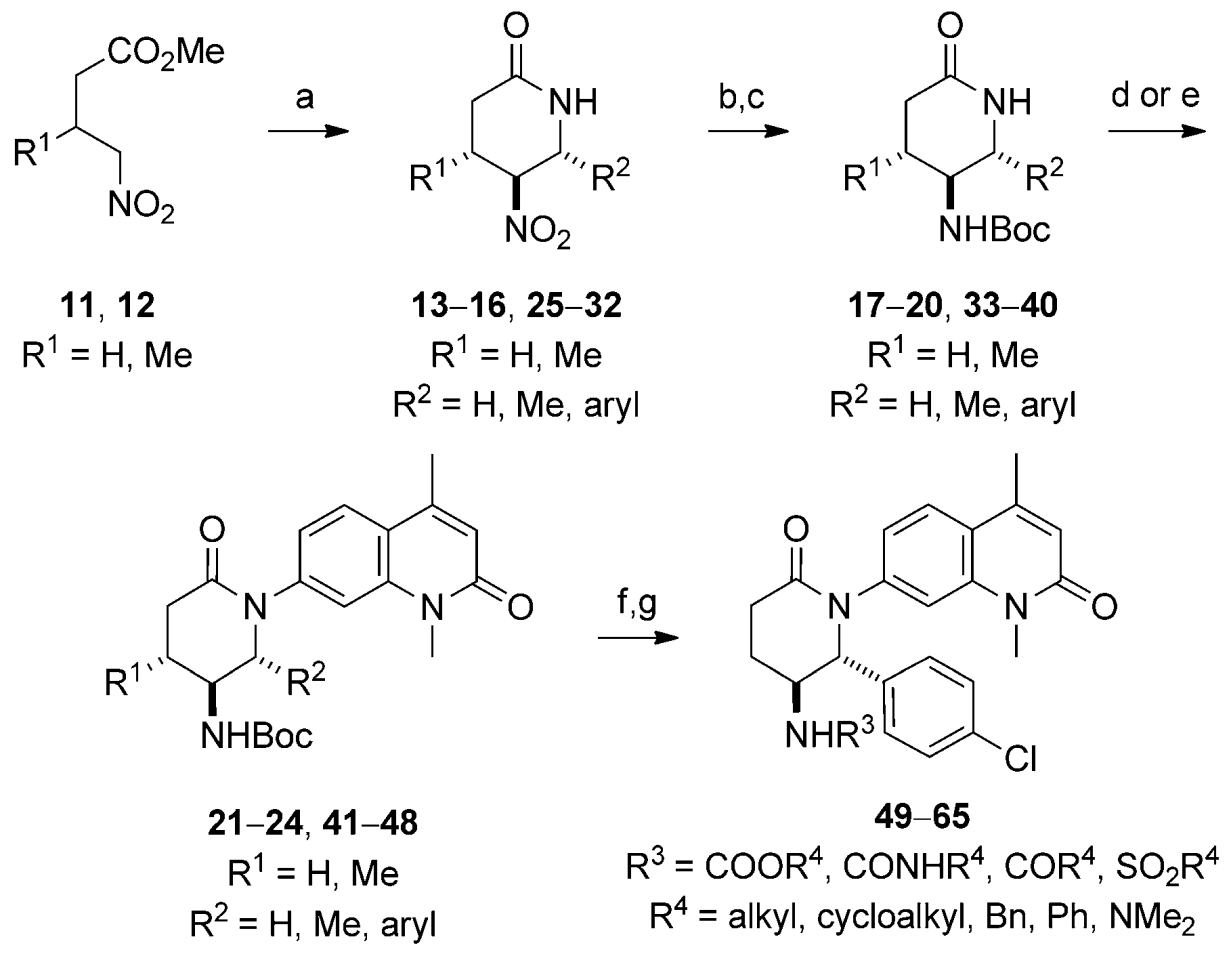
Synthesis of analogues for SAR studies around the lead scaffold. Reagents and conditions: a) R^3^CHO, NH_4_OAc, EtOH, 90 °C, 26–86 %, d.r. 2:1–>20:1; b) NiCl_2_⋅6 H_2_O, NaBH_4_, MeOH, 0 °C; c) Boc_2_O, 39–91 % (2 steps); d) 4, [Pd_2_(dba)_3_], Xantphos, Cs_2_CO_3_, 1,4-dioxane, 100 °C, 2–75 %; e) 4, K_3_PO_4_, CuI, (±)-*trans*-1,2-diaminocyclohexane, 1,4-dioxane, 97 °C, 7–65 %; f) HCl/dioxane, 96 %; g) R^2^Cl, TEA, CH_2_Cl_2_ or RNCO, CH_2_Cl_2_, 12–60 %. Boc=*tert*-butyloxycarbonyl, TFA=trifluoroacetic acid.

The co-crystal structure suggested that potency could be further boosted by additional substitution of the newly installed aryl ring and alternative derivatization of the amine to optimize H bonding to G43 and hydrophobic interactions with F47. Halogenated, methylated, and methoxylated benzaldehydes were used in the nitro-Mannich/lactamization cascade process (**25**–**32**) and carried through to the *N*-Boc-protected lactams **33**–**40** as before. As the C6-aryl-substituted lactams gave exceedingly low yields in the Buchwald–Hartwig coupling (2 %), a Cu-mediated Goldberg coupling was used instead to furnish the coupled products **41**–**48** in acceptable yields.^15^ All substituents, except a *para*-methoxy group, were well tolerated, with **48** (R^1^=H, R^2^=*p*-Cl-C_6_H_4_) being the strongest binder according to DSF analysis (Δ*T*_m_=4.4±0.72 °C; Table S1).

Variations to the substituent on the amino group were more productive in improving binding to BRD9. The carbamate protecting group was removed with HCl/dioxane, and the resulting amine was derivatized through reactions with a range of acyl chlorides, chloroformates, isocyanates, and sulfonyl chlorides to give various amides, carbamates, ureas, and sulfonamides for testing (**49**–**65**; see Table S1). No single chemical class dominated binding; the four best compounds, **48**, **55** (R^3^=Bz), **60** (R_3_=SO_2_*i*Bu), and **64** (R^3^=CONHPh), all possessed different functional groups.

Towards the asymmetric synthesis of the most active compounds, various organocatalysts were trialed in the nitro-Mannich reaction of **11** with imine **66** to obtain the desired enantiomer of **67** in a selective fashion (Scheme 3). Of these, recently developed bifunctional cinchona-alkaloid-derived phase-transfer catalysts were found to impart the highest enantiofacial selectivity.^16^ Following optimization, quinidine-derived catalyst **68** furnished the desired product **67** on gram scale as a 7:1 mixture of diastereomers, both in 90 % *ee. N*-Boc deprotection and concomitant cyclization with TFA, followed by epimerization with DBU, afforded lactam **69** as a single diastereomer. The synthesis was then completed as before to give compounds **48**, **55**, **60**, and **64** in 90 % *ee*, with further purification by preparative HPLC on a chiral stationary phase providing these compounds in >98 % *ee*. ITC analysis of these compounds revealed (2*R*,3*S*)-**60**, hereafter referred to as **LP99**, to be the most potent compound synthesized, with a *K*_D_ value of 99 nm against BRD9 (Table 2). This binding was entirely driven by enthalpic interactions (Δ*H*=−11 kcal mol^−1^), with a net loss in entropy upon binding (*T*Δ*S*=−2.0 kcal mol^−1^), which is consistent with a number of specific interactions and may offer an advantage for wider selectivity.^17^ The importance of chirality and configuration in this work is highlighted by the fact that the enantiomer of **LP99** showed no detectable binding to BRD9 by ITC (Figure S8).

**Scheme 3:**
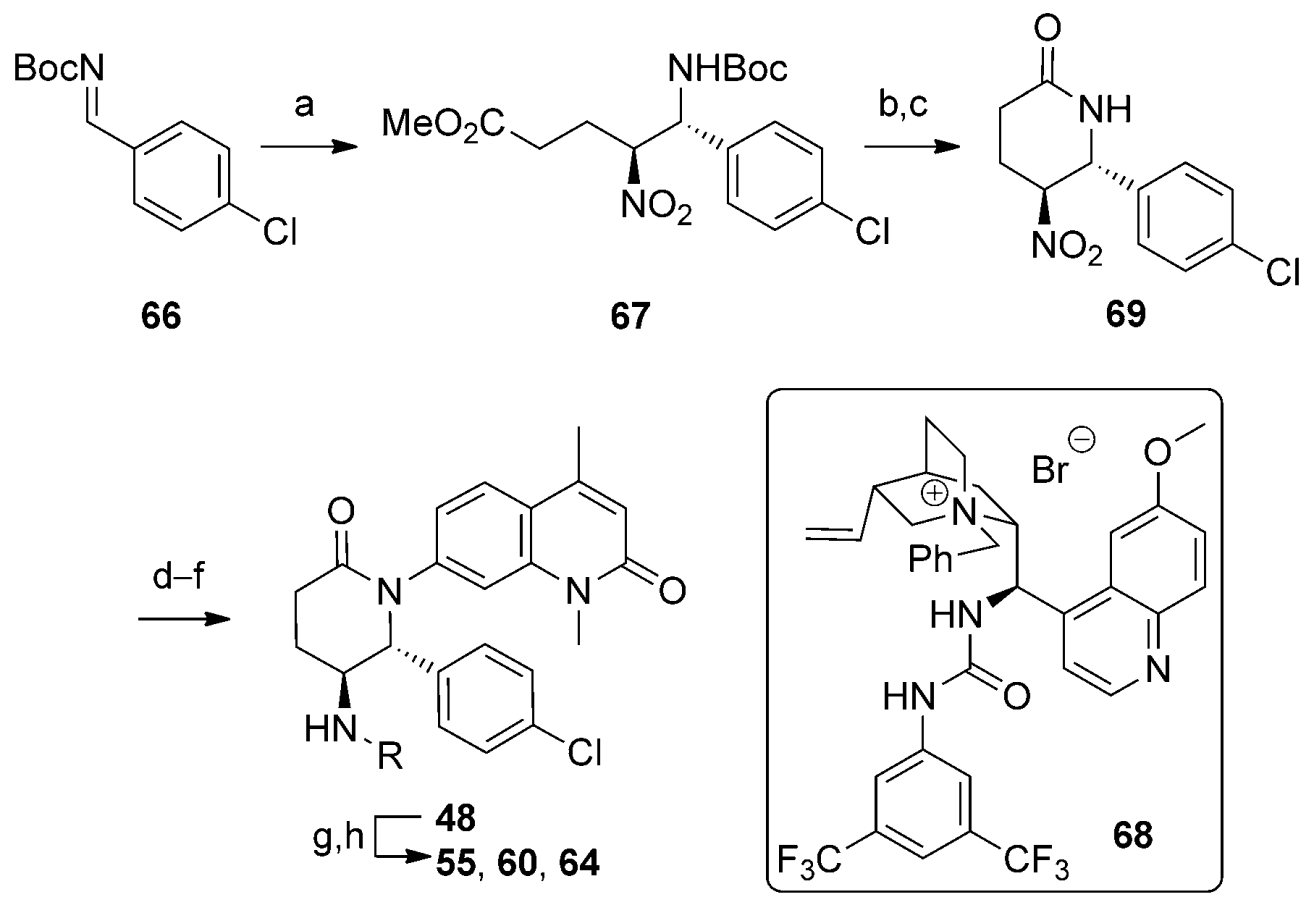
Organocatalytic enantioselective synthesis of BRD9 inhibitors. Reagents and conditions: a) 11, K_2_CO_3_, 68 (10 mol %), TBME, −20 °C, 70 %, d.r. 7:1, *ee*_major_ 90 %/*ee*_minor_ 90 %; b) TFA, CH_2_Cl_2_; c) DBU, CH_2_Cl_2_, 73 % (2 steps); d) NiCl_2_⋅6 H_2_O, NaBH_4_, MeOH, 0 °C; e) Boc_2_O, 74 % (2 steps); f) 4, K_3_PO_4_, CuI, (±)-*trans*-1,2-diaminocyclohexane, 1,4-dioxane, 97 °C, 65 %; g) HCl/dioxane, 96 %; h) RCl, TEA, CH_2_Cl_2_ or RNCO, CH_2_Cl_2_, 25–40 %. DBU=1,8-diazabicyclo[5.4.0]undec-7-ene, TBE=*tert*-butyl methyl ether, TEA=triethylamine.

**Table 2:** BRD9 potency of (2*R*,3*S*) derivatives by ITC.

	Compound	R	*K*_D_ [nm]	Δ*H* [kcal mol^−1^]	*T*Δ*S* [kcal mol^−1^]
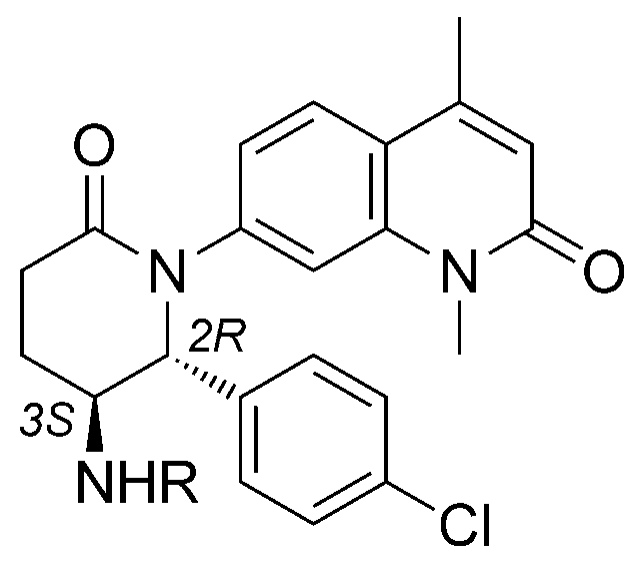	**48**	Boc	247	−9.88	1.17
	**55**	Bz	2000	−8.06	−0.764
	**60**	SO_2_*i*Bu	99	−11.2	1.98
	**64**	CONHPh	1010	−6.52	−1.39

Bz=benzoyl.

Inhibitor **LP99** was further assessed in a number of biological assays. This compound was profiled broadly for BRD selectivity by DSF (Figure 3) against all expressible BRDs (48 of 61 in the human genome), showing exquisite selectivity with <1 °C stabilization of all BRDs, including other members of sub-family IV, except BRD7/9 (Figure 3).

**Figure 3:**
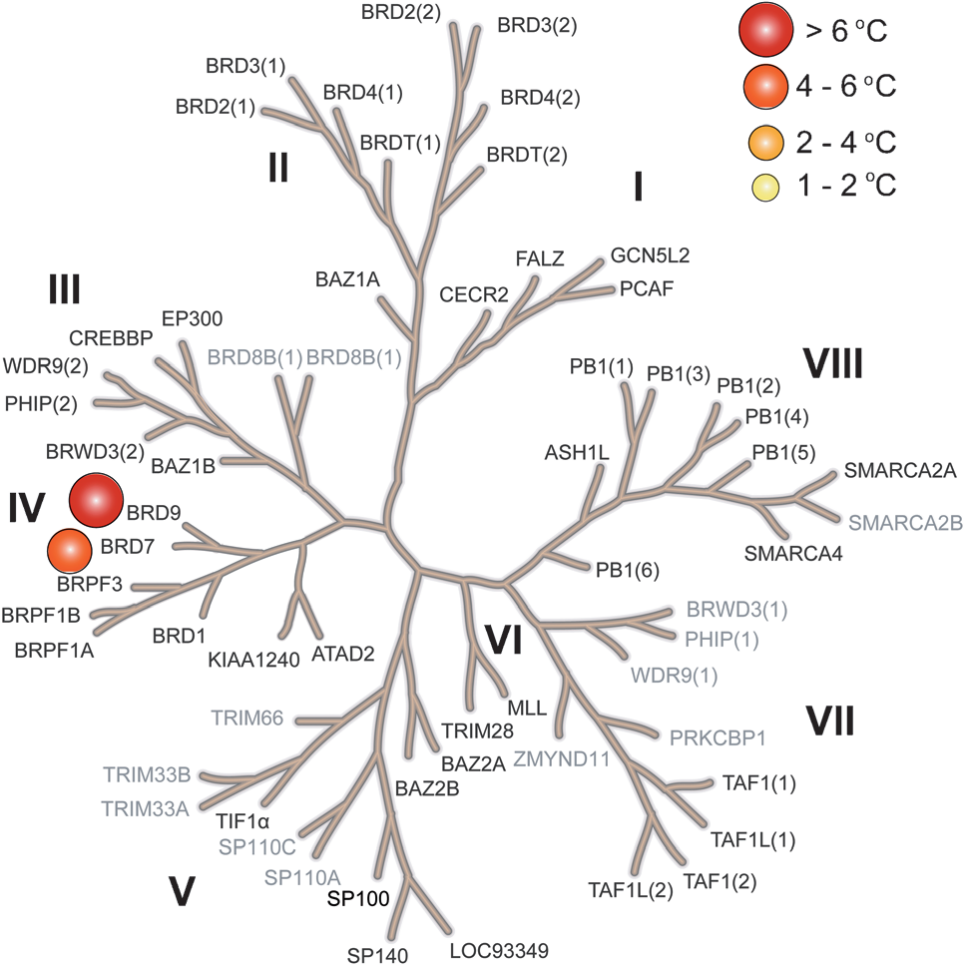
LP99 is a potent and selective BRD7/9 inhibitor. Selectivity panel of LP99 against 48 BRDs (bold type) at 10 μm in terms of the Δ*T*_m_ values determined by DSF.

Inhibition of BRD7/9–histone interactions in cell assays was also examined. The cellular efficacy of **LP99** on BRD9 was investigated using a fluorescence recovery after photobleaching (FRAP) assay (Figure S10):^18^ **LP99** was found to disrupt BRD9 interactions with chromatin at a concentration of 0.8 μm. To measure this further, a bioluminescence resonance energy transfer (BRET) assay was performed. BRD7– and BRD9–NanoLuc luciferase fusion proteins and fluorescently labelled histone H3.3– and H4–HaloTag fusions were expressed in HEK293 cells.^19^ The addition of **LP99** decreased BRET for both BRD7 and BRD9 in both the H3.3 and H4 systems in a dose-dependent manner, with cellular IC_50_ values in the low micromolar range for both histones (Figure 4 A; see also Figure S11 and Table S3). Taken together, these cellular assays demonstrate that the BRD7/9 inhibitor **LP99** is able to disrupt the binding of BRD7 and BRD9 to chromatin in cells. Furthermore, cytotoxicity tests in U2OS cells for 24 and 72 hours showed the inhibitor to be non-toxic at concentrations of <33 μm (Figure S12).

**Figure 4:**
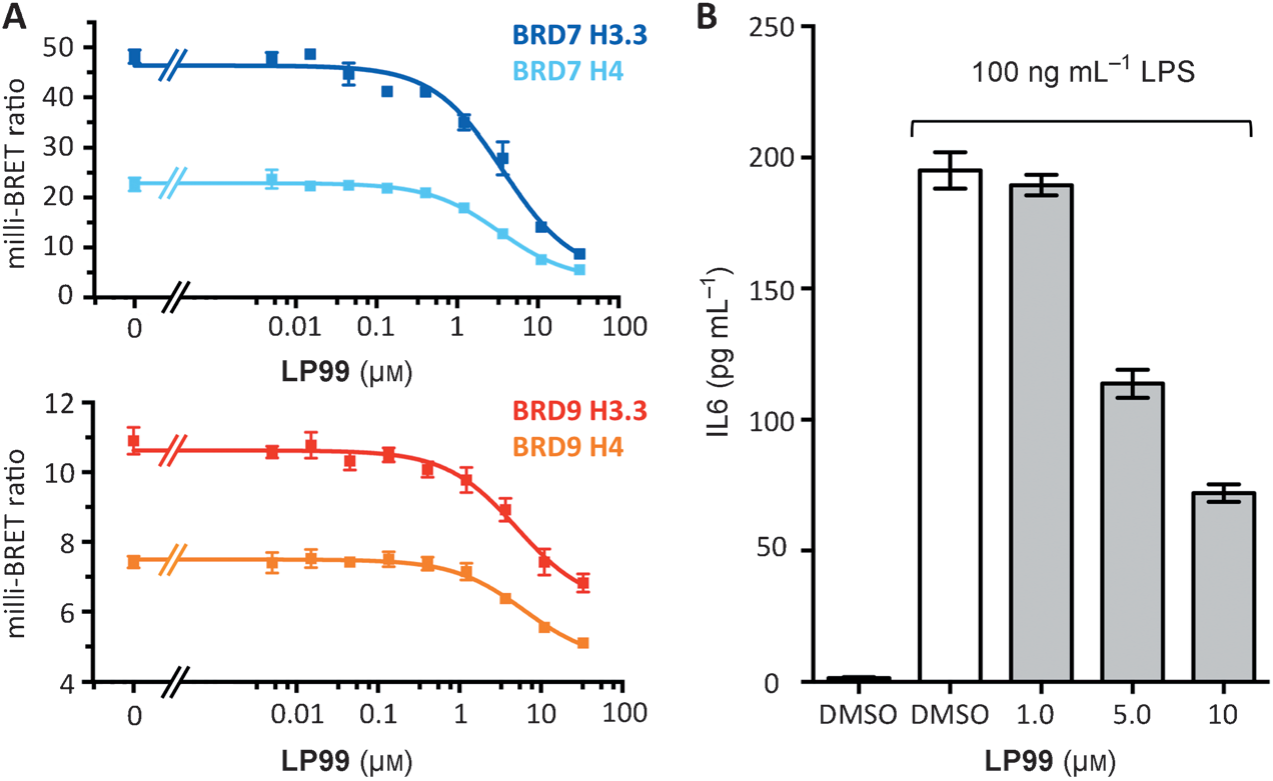
LP99 is active in cellular assays. A) BRET assay of LP99 on BRD7 and BRD9 fusion proteins. B) LP99 inhibits the expression of IL6 in LPS-stimulated THP-1 cells.

To investigate if BRD7/9 could influence the expression of pro-inflammatory cytokines, a human THP-1 monocytic cell line was stimulated with lipopolysaccharide (LPS), and the influence of **LP99** on the secretion of interleukin 6 (IL-6) was measured by an enzyme-linked immunosorbent assay (ELISA; Figure 4 B). **LP99** inhibited IL-6 secretion from THP-1 cells in a dose-dependent manner, demonstrating that BRD7/9 BRDs are potential targets for anti-inflammatory treatment. The effect of **LP99** on IL-6 expression demonstrates for the first time that a small-molecule BRD7/9 inhibitor may have a similar function and utility to IL-6 neutralizing antibodies, such as tocilizumab, in the treatment of rheumatoid arthritis.^20^

In conclusion, by using a structure-based design approach, the simple BRD binding fragment **1** has been developed into **LP99**, a potent and selective inhibitor of the closely related BRDs of BRD7 and BRD9. Incorporating tractable chemical synthesis, through a nitro-Mannich/lactamization cascade and the use of a bifunctional cinchona-alkaloid-derived phase-transfer catalyst, allowed for rapid establishment of structure–activity relationships and access to the lead enantioenriched scaffold on scale.

The use of ligand–protein co-crystallography was crucial to determine the preferred absolute configuration of the ligands and in the design and synthesis of analogues with increased potency. The selectivity of the most potent analogue, **LP99**, was extensively characterized, and the compound was shown to inhibit only two of the 48 BRDs. Furthermore, this compound was shown to disrupt the association of tagged BRD7 and BRD9 constructs from both bulk chromatin (by a FRAP assay) and individual histone proteins (by a NanoBRET assay). We have shown for the first time that aside from the BRM and BRG catalytic subunits of the SWI/SNF complex, BRD7 and BRD9 also play a role in the regulation of inflammatory cytokines and are potential novel targets for anti-inflammatory treatment. As the first potent, selective, and cell-active inhibitor of BRD7/9, **LP99** will serve as a valuable tool in further deciphering the biological roles of these important BRD-containing proteins and serve as a starting point in the discovery of a new class of epigenetic therapeutics.

## Supporting Information

As a service to our authors and readers, this journal provides supporting information supplied by the authors. Such materials are peer reviewed and may be re-organized for online delivery, but are not copy-edited or typeset. Technical support issues arising from supporting information (other than missing files) should be addressed to the authors.

miscellaneous_information
